# The Implementation of Patient-Centered Humanistic Care for COVID-19 Closely Contacted Hemodialysis Patients Under the Hospital-Based Group Medical Quarantine: A Brief Research Report

**DOI:** 10.3389/fpsyg.2021.553234

**Published:** 2021-10-08

**Authors:** He Zhang, Liangying Gan, Xiaodan Li, Xiaofeng Shao, Li Zuo, Jie Gao, Xiaobo Huang, Xiaojun Jia, Junqing Liang, Zhihua Hou, Yanhua Wang, Lei Wang, Zhancheng Gao, Jianliu Wang, Hongsong Chen

**Affiliations:** ^1^Administration Office, Peking University People's Hospital, Beijing, China; ^2^School of International Studies, Renmin University of China, Beijing, China; ^3^Department of Nephrology, Peking University People's Hospital, Beijing, China; ^4^Department of Nursing, Peking University People's Hospital, Beijing, China; ^5^Department of Publicity, Peking University People's Hospital, Beijing, China; ^6^Department of Hepatobiliary Surgery, Peking University People's Hospital, Beijing, China; ^7^Urology and Lithotripsy Center, Peking University People's Hospital, Beijing, China; ^8^Department of General Affairs, Peking University People's Hospital, Beijing, China; ^9^Department of Trauma and Orthopedics, Peking University People's Hospital, Beijing, China; ^10^Department of Respiratory and Critical Care Medicine, Peking University People's Hospital, Beijing, China; ^11^Department of Obstetrics and Gynecology, Peking University People's Hospital, Beijing, China; ^12^Peking University People's Hospital, Beijing, China; ^13^Peking University Hepatology Institute, Beijing, China

**Keywords:** humanistic care, measures, COVID-19, hospital-based group medical quarantine, hemodialysis patients

## Abstract

In February 2020, an inpatient in Peking University People's Hospital (PKUPH), China, was confirmed positive for the novel coronavirus. In this case, 143 hemodialysis patients were labeled as close contacts and required to be placed under the hospital-based group medical quarantine (HB-GMQ) for 2 weeks by the authorities. After the case was reported, false or misleading information about the case flourished on social media platforms, which led to infodemic. Under this context, PKUPH adopted patient-centered humanistic care to implement the HB-GMQ, through the synergy of administrative, healthcare, logistical, and other measures under the model of patient-centered care of the Massachusetts Medical Society (MMS). As a result, all the patients tided over the HB-GMQ with no COVID-19 infection and no unanticipated adverse events, and all met the criteria for lifting the HB-GMQ. According to the questionnaires taken during the HB-GMQ, a high level of satisfaction was found among the quarantined and no symptomatic increase of anxiety and depression in the patients before and during the HB-GMQ, by comparing the Zung self-rating anxiety scale (SAS) and self-rating depression scale (SDS) conducted in December 2019 and on the 12th day of the HB-GMQ. This article is to brief on PKUPH's experience in implementing patient-centered humanistic care tailored to hemodialysis patients under the HB-GMQ, and to validate the hypothesis that patient-centered humanistic care is effective and helpful to help them tide over the HB-GMQ, so as to shed light on how to implement the HB-GMQ and cope with the HB-GMQ-induced problems in other hospitals.

## Introduction

Humanism in medicine is fundamental to excellent patient care (Chou et al., 2014), which is an aspect imperative to quality healthcare (Muneeb et al., 2017). Generally, humanistic care is referred to as a patient-centered approach emphasizing a positive and trusting relationship between the caregiver and a patient, characterized by collaboration, dignity, empathy, and trust. In 1988, the Picker Institute in Britain coined the term “patient-centered care” to call attention to the need for healthcare practitioners and systems to provide care not only from a clinical perspective, but also from the patient and family perspective (Gerteis et al., 1993). In 2001, the Institute of Medicine defined patient-centered care as “providing care that is respectful of, and responsive to, individual patient preferences, needs and values, and ensuring that patient values guide all clinical decisions” (Institute of Medicine Committee on Quality of Health Care in America., 2001). In this sense, when patient-centered approach and attitude are adopted, patients are partners with healthcare practitioners who will focus more on emotional, mental, spiritual, social, and financial needs of patients, rather than just on their clinical needs. And patient-centered care can be achieved by devoting visit time to non-medical aspects of the life of the patients, maximizing quality face-to-face time in patient interaction, empathizing with the patient, and involving the patients in decisions about their care (Draeger and Stern, 2014).

A large body of research supports the hypothesis that patient-centered humanistic care is fundamental to excellent patient care. Everhart et al. (2019) believed that “patient-centered care and patient- and family-centered care (PFCC) improve patient and family satisfaction, patient self-management, and physical and mental health outcomes.” Delaney (2017) reiterated the benefits of humanistic medical care and stressed that the patient-centered approach, as a primary approach to healthcare, can enhance healthcare, and patient satisfaction.

The outbreak of the novel coronavirus, which began in 2019, has become a global pandemic and posed a significant threat to global health. To resist the spread of the virus, quarantine and isolation are deemed as critical measures that have been put in place across the globe. On February 18, 2020, an inpatient in Peking University People's Hospital (PKUPH) was confirmed positive for the novel coronavirus, leading to hospital-based group medical quarantine (HB-GMQ) of 143 hemodialysis patients for 2 weeks.

Unlike putting healthy people under the HB-GMQ, it was challenging for PKUPH to take care of vulnerable hemodialysis patients in terms of healthcare, psychology, dietary, etc. First, most hemodialysis patients are elderly people who have more or less psychological disorders, underlying diseases, and comorbid conditions; delicate care and emergency preparedness should always be considered in the workplace for any unexpected event. Second, hemodialysis patients are subjected to unusual emotional stress and have a wide variety of psychological problems (Levy, 1979). All hemodialysis patients were in a state of distress and worried about the possibility of infection. Third, quarantine might have further aggravated their preexisted psychological problems, as it often comes with distressing side effects, including post-traumatic stress disorder (PTSD). Fourth, following a kidney-friendly diet further increased the difficulty of providing care to these patients, as dietary requirements differ from patient to patient depending on the state of the condition. Faced with all these challenges, PKUPH, in adherence to COVID-19 protocols and recommendations and advice for the healthcare settings and public by the World Health Organization (WHO) (2020) and the Chinese health authorities, adopted a patient-centered humanistic approach and attitude to implement the HB-GMQ under the model of patient-centered care of the Massachusetts Medical Society (MMS).

Although the outbreak of infectious diseases such as SARS, MERS, EBOLA, and COVID-19 stimulated related research, there still remains little research on the psychological impact on hemodialysis patients and how to implement the HB-GMQ for those who are directly exposed to infectious diseases. Therefore, the purpose of this study was to validate the hypothesis that patient-centered humanistic care is effective in reducing anxieties or depression in hemodialysis patients, increasing their satisfaction and compliance with the HB-GMQ.

## Methods

### Setting and Sample

After the case was reported, 143 hemodialysis patients were labeled as close contacts and required to be quarantined by the authorities. PKUPH, under the guidance of the governments at different levels, immediately set up a working group to manage the HB-GMQ and adopted patient-centered humanistic care approach to help these patients safely tide over the quarantine. The study was performed on a designated campus called Baitasi Campus for a 14-day period since the quarantine had been imposed on February 18, 2020. The inclusion criteria were as follows: (1) regular hemodialysis ≥3 months; (2) under the HB-GMQ; and (3) informed consent and signed informed consent form. The exclusion criteria were as follows: (1) acute blood loss or acute infection within 3 months; (2) cardiovascular and cerebrovascular accidents, rheumatic immune diseases, or uncontrolled malignant tumors occurred within 3 months; (3) people with mental illness; and (4) people with severe cognitive impairment. And the withdrawal criteria were as follows: (1) patients who failed to complete the HB-QMG and (2) withdrawal from the study upon the request of subjects.

Before the quarantine, the nucleic acid amplification test (NAAT) was given to the close contacts and the results were all negative. During the quarantine, the patients received maintenance hemodialysis as they did in the past. After 14 days, patients with no clinical symptoms of COVID-19 and no severe health conditions could be discharged.

At the initial phase of the HB-GMQ, the demographic characteristics of conditions of the patients were sorted out from medical records. With the information, medical staff could make informed decisions, pay special attention to those with severe conditions, provide expert medical consultations, and render individualized care accordingly.

### Patient-Centered Humanistic Care Provided by PKUPH During HB-GMQ

Leaving a familiar and comfortable home environment for the HB-GMQ would be difficult for the most hemodialysis patients. The call for the HB-GMQ would result in anxieties or distress on the patients, which in turn would project undesirable emotions onto someone else and hamper the implementation of the HB-GMQ. Initially, two patients in this case were unwilling to be quarantined for one reason or another. Persuaded by the communities and the local governments, they finally accepted the HB-GMQ. In order to stabilize the emotions of the patients, prevent their symptomatic increase of anxiety and depression, avoid their misunderstandings toward quarantine arrangements, and help them smoothly tide over this special period, PKUPH formed a working group and incorporated patient-centered humanistic care into the day-to-day routine management, healthcare, and logistics, trying its best to provide convenience as much of the home environment as possible. During the HB-GMQ, the working group put patients first as the core values and aimed to achieve the objectives of “no COVID-19 infection and no unanticipated adverse events.”

**Figure 1:**
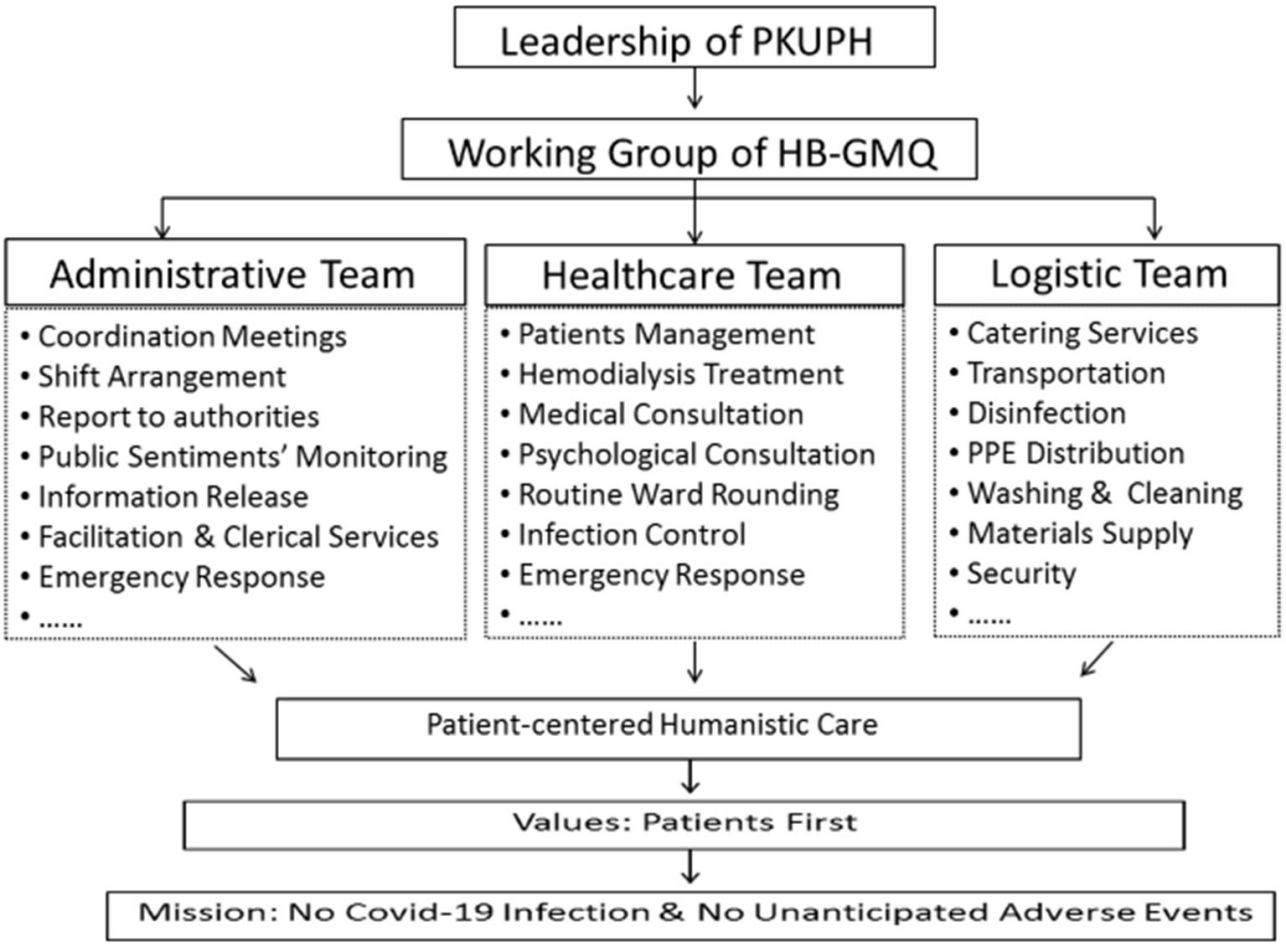
Organizational arrangement of PKUPH working group of HB-GMQ.

A working group led by two vice presidents of PKUPH was soon established after the case was confirmed. Heads from different executive departments and medical specialists from different fields of practice were mobilized to establish a coordination mechanism (Figure 1). In accordance with COVID-19 protocols and guidelines of the WHO and Chinese health authorities, PKUPH provided patient-centered care under the model of patient-centered care of the MMS, which is presented in Table 1.

**Table 1:** PKUPH's patient-centered humanistic care under the model of MMS.

**Indicators under the model of patient-centered care of the MMS**	**PKUPH's approach**	
Mission and values aligned with patient goals	PKUPH put the quarantined hemodialysis patients first, with the mission of “no COVID-19 infection and no unanticipated adverse events during the HB-GMQ.”	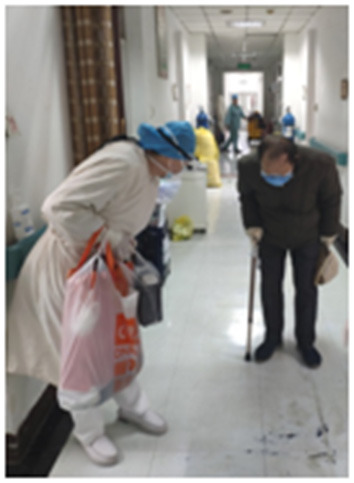
Care is collaborative, coordinated, accessible	During the HB-GMQ, routine dialysis treatment, expert medical consultations, and routine ward rounds were provided. Nephrologists and nurses were available 24 h, and a multidisciplinary team composed of specialists from different fields of practice was established to meet and respond to the healthcare needs of the patients during the HB-GMQ. Moreover, relatively fixed positioning of medical staff was arranged in the hope that all medical staff can grasp all the information and the characteristics of each patient to avoid poor information sharing due to handovers.	
Psychological comfort and emotional well-being are top priorities	Nephrologists and nurses at PKUPH were required to pay attention to the physical and mental well-being of the patients during rounding, and render timely psychological counseling and emotional support according to patient-specific symptoms and psychological survey scale. Second, PKUPH provided health education about the coronavirus for the quarantined during the HB-GMQ aiming to increase their awareness of prevention and relieve their worries about the virus. Third, nephrologists and nurses at PKUPH increased the frequency of daily visit and created a group chat on social network APP to collect the claims of the patients and timely responding to their requests, such that strengthened the communication between the patients and improved the patients-medical staff relations. Fourth, to follow a renal diet, the catering staff tried every means to come up with a diet that was tailored to the needs of the patients, and met their individualized diet needs under the guidance of nephrologists and nutritionists. Fifth, the working group connected with local authorities to solve the issuing of the quarantine pass, formulate return-to-community plan, and tackle the problem of community inaccessibility.	
Family welcome in care setting	Each patient was allowed to bring a family member to live with them during the HB-GMQ. Their family members were not allowed to visit home and return back to the hospital during the HB-GMQ period.	
Patient and family always included in decisions	Treatments and therapies were provided based on the discussions with the patients and family.	
Patient and family viewpoints respected and valued	Respected and valued the patients and their family's decisions and collaborated to facilitate the HB-GMQ-induced problems.	

*NEJM (2020). NEJM Catalyst (catalyst.nejm.org) © Massachusetts Medical Society*.

*The left column of this table explains the model of patient-centered care of the MMS, and the middle column of this table describes PKUPH's approach under the model of patient-centered care of the MMS. The picture in the right column of this table shows a 72-year-old hemodialysis patient bowed to the working staff to show his gratitude to the care he received during the HB-GMQ. Photographed by Lian Zu (Health News, 2020)*.

### Instruments

During the HB-GMQ, PKUPH practiced patient-centered humanistic care through the synergy of administrative, medical, logistical, and other specific measures under the model of patient-centered care of the MMS. The model of patient-centered care of the MMS involves seven indicators: (1) mission and values aligned with patient goals; (2) full transparency and fast delivery of information; (3) care is collaborative, coordinated, accessible; (4) family welcome in care setting; (5) psychological comfort and emotional well-being are top priorities; (6) patient and family always included in decisions; (7) patient and family viewpoints respected and valued. The PKUPH's patient-centered humanistic care under the model of patient-centered care of the MMS is presented in “Patient-centered Humanistic Care Provided by PKUPH during HB-GMQ” section.

In order to assess the effectiveness of the PKUPH's approach of patient-centered humanistic care, electronic satisfaction questionnaires were administered by nurses in charge to 143 hemodialysis patients to fill out on the 14th day of the HB-GMQ, and the differences in hemodialysis patients' anxious or depressive symptomatology before and during the HB-GMQ were recorded by the Zung self-rating anxiety scale (SAS) and self-rating depression scale (SDS) conducted in December 2019 and on the 12th day of the HB-GMQ.

The patient satisfaction survey investigated seven aspects of satisfaction: (1) hospital meals, (2) living environment, (3) dialysis treatment, (4) transfer services, (5) hospital hygiene, (6) material supply, and (7) overall experience of the HB-GMQ. The level of satisfaction was divided into five categories: very satisfactory, satisfactory, ordinary, unsatisfactory, and very unsatisfactory. To avoid potential source of bias, data of “unsatisfactory” and “very unsatisfactory” were also recorded in the study. The detailed information is presented in the “Result” section.

The Zung SAS and SDS are methods of measuring levels of anxiety and depression in patients who have anxiety-related and depression-related symptoms. Both are self-administered surveys with 20 questions on the scale. Under the SAS and SDS, each response was scored on a scale of 1–4 (none or a little of the time, some of the time, a good part of the time, most or all of the time). Under the SAS, the authors divided the rough score by 80 to get the anxiety severity index scores, which range from 0.25 to 1.00, with 0.50–0.59 in Mild Anxiety Levels; 0.60–0.69 in Moderate to Severe Anxiety Levels; 0.70 and above in Severe Anxiety Levels. Under the SDS, the authors divided the rough score by 80 to get the depression severity index scores, which range from 0.25 to 1.00, with 0.50–0.59 in Mildly Depressed; 0.60–0.69 in Moderately to Severely Depressed; 0.70; and above in Severely Depressed (Wu, 2015).

### Procedures

The best way of measuring whether the practice was patient-centered or not lies in the assessment made by the patients themselves. To evaluate the effectiveness of the implementation of patient-centered humanistic care practiced by PKUPH during the HB-GMQ, satisfaction questionnaires were administered to 143 hemodialysis patients on the 14th day of the HB-GMQ by nurses in charge (Table 2).

**Table 2:** Data of patient satisfaction questionnaire.

**Satisfaction survey questions**	**Very satisfactory**	**Satisfactory**	**Ordinary**	**Unsatisfactory**	**Very unsatisfactory**
Are you satisfied with the hospital's meals?	28 (21.54%)	48 (36.92%)	39 (30%)	9 (6.92%)	6 (4.62%)
Are you satisfied with the room you are in?	32 (24.62%)	59 (45.38%)	28 (21.54%)	8 (6.15%)	3 (2.31%)
Are you satisfied with the dialysis treatment process?	84 (64.62%)	45 (34.62%)	1 (0.77%)	0	0
Are you satisfied with the hygiene of the hospital?	56 (43.08%)	59 (45.38%)	10 (7.69%)	4 (3.08%)	1 (0.77%)
Are you satisfied with the transfer services during the dialysis process?	70 (53.85%)	52 (40%)	4 (3.08%)	2 (1.54%)	2 (1.54)
Are you satisfied with material supply of the hospital?	60 (46.15%)	65 (50%)	4 (3.08%)	0	1 (0.77%)
Your overall satisfaction with the care during GMQ period?	51 (39.23%)	64 (49.23%)	10 (7.69%)	2 (1.54%)	3 (2.31%)

**Figure 2:**
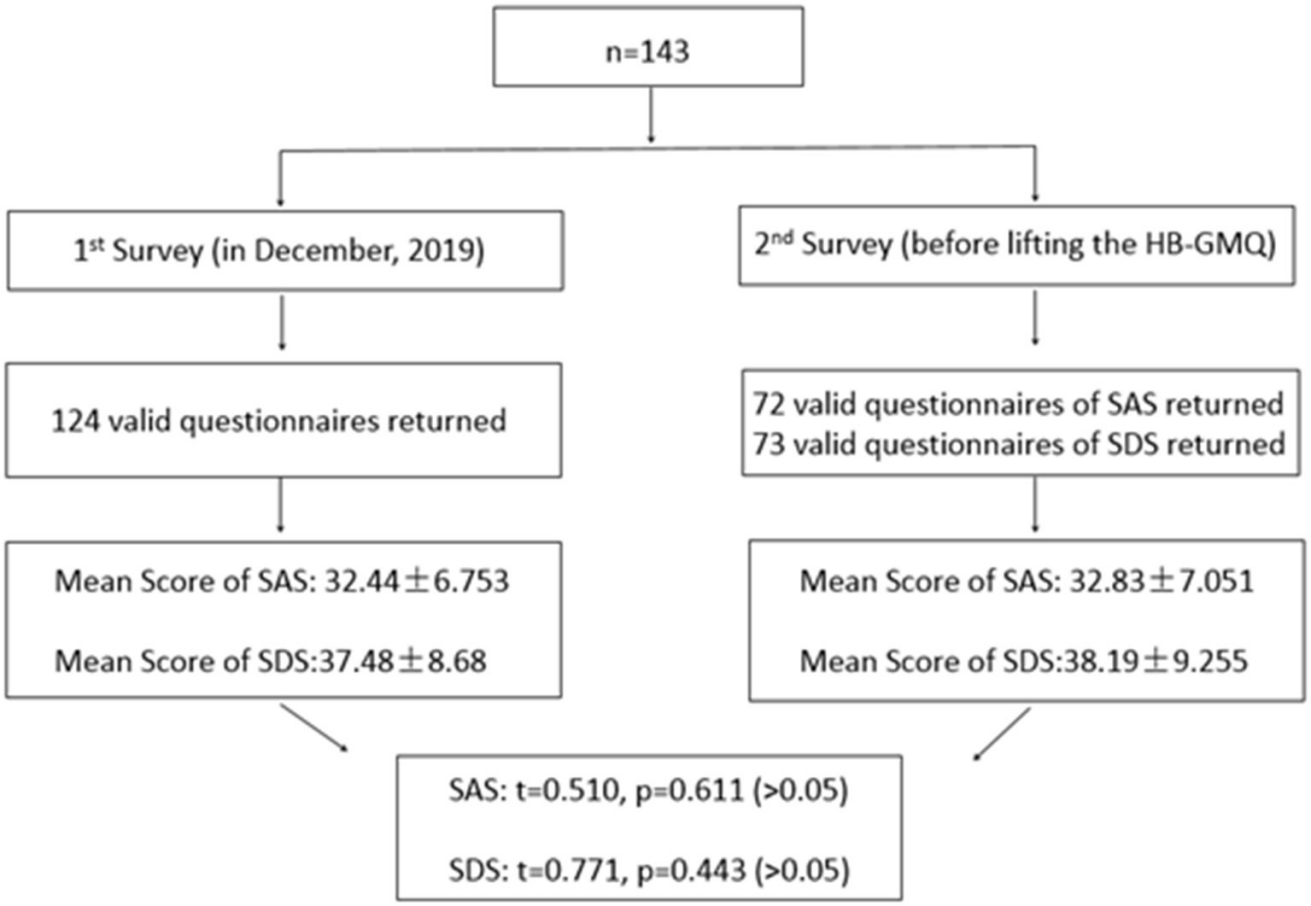
SAS and SDS scores of study respondents.

At the Hemodialysis Center of PKUPH, it is a routine job for nurses to collect the patients' data of SAS and SDS on a regular basis to evaluate the level of anxiety and depression. To compare the differences in the anxious or depressive symptomatology of the patients before and during the HB-GMQ, the data of SAS and SDS that were obtained in December 2019 were used to compare with the data that were collected on the 12th day of the HB-GMQ in 2020 (Figure 2).

All the above-mentioned procedures were conducted after the patients signed the consent form. And those who were involved in the data collection of SAS and SDS in December 2019 provided written consent to join the study during the HB-GMQ in 2020. For those who were not convenient to fill out the questionnaires during maintenance dialysis, the nurses in charge shall help them fill out the questionnaires.

### Statistical Analyses

In the analysis of the satisfaction survey, categorical variables were presented as numbers with percentages. In the analyses of SAS and SDS, differences in the anxious or depressive symptomatology of the patients before and during the HB-QMQ were examined by using a paired *t*-test. The statistical significance level was set at 0.05. The hemodialysis management system (Copyright 2006, Beijing Whole Way Technology Co. Ltd) was used for data entry and scoring. Other data were entered into an EXCEL worksheet. The SPSS software package (version 22.0, SPSS Inc., Chicago, IL, United States) was used for statistical analyses.

## Results

### Demographic Characteristics of Participants

A total of 143 hemodialysis patients were enrolled in the study. Of them, 94 are male patients (65.7%), and 49 are female (34.3%). The median age of them was 59.5 years, ranging in age from 30 to 93. The proportion of those over 60 years reaches 53.1%, of which one case is >90 years, 13 cases are 81–90 years, 24 cases are 71–80 years, and 38 cases are 60–70 years.

After combining through the medical records of these 143 hemodialysis patients, at least 75 patients (52.4%) had underlying diseases and comorbidities, 44 patients (35%) had a history of cardiovascular and cerebrovascular diseases, and 22 patients had a history of cancer (15.5%), with cirrhosis in two cases and gastrointestinal ulcer in one case. Twenty-five cases with severe illness were screened out among the 143 patients, and their information was fully shared by the medical staff. In addition, 24 patients had their relatives accompanied during the HB-GMQ.

### Patient Satisfaction About HB-GMQ

On the 14th day of the HB-GMQ, electronic satisfaction questionnaires were administered to 143 hemodialysis patients, and 130 valid questionnaires were returned. About 99.23% of the respondents agreed that timely solutions were provided when they need help; 96.92% believed that medical staff responded to their questions concerning medical or living needs; 99.23% agreed that the healthcare team assisted them with meal delivery, water fetching, etc. Overall, 115 patients gave very satisfactory or satisfactory (88.46%), 10 patients gave ordinary (7.69%), and five patients gave unsatisfactory (3.85%) (Table 2). Among the unsatisfactory patients, the main reason for “unsatisfactory” was due to the lack of freedom caused by the HB-GMQ.

### Comparison of SAS and SDS Scores Before and During HB-GMQ

In December 2019 (before the HB-GMQ), mental health of 124 cases was effectively assessed by using SAS and SDS, which is a routine job for the Hemodialysis Center of PKUPH. On the 12th day of the HB-GMQ in 2020, SAS and SDS questionnaires were sent to 143 patients. The valid sample size that can be self-paired in the data before and during the HB-GMQ was 72 in SAS and 73 in SDS.

In the study, the authors found that there were no statistically significant differences in anxious or depressive symptomatology of the patients before and during the HB-GMQ by comparing the SAS and SDS scores assessed before the HB-GMQ with those assessed during the HB-GMQ (Figure 2).

## Discussion

Our study showed that a high level of satisfaction about the HB-GMQ was found among hemodialysis patients. This was mainly due to the successful implementation of patient-centered humanistic care for the quarantined. In order to ensure the smooth execution of the HB-GMQ, 32 coordination meetings were organized, 11 medical and nursing regulations and processes were formulated, 2,292 N95 masks and 11,100 surgical masks were consumed, 10,794 sets of meals were offered, and 265 staff members were on site providing services to the quarantined during the HB-GMQ.

During the entire process of the HB-GMQ, patient-centered humanistic care played a crucial role in the successful implementation of the HB-GMQ and in the achievement of the following results: First, patient-centered humanistic care could attenuate negative and reluctant reactions of patients to the HB-GMQ, and gain cooperation and understanding of the patients about the HB-GMQ. Second, patient-centered humanistic care could help build a positive and trusting relationship between the patients and healthcare practitioners during the HB-GMQ. Third, patient-centered humanistic care could alleviate the anxieties or worries of the patients who already suffered from psychological disorders or emotional instabilities caused by the kidney disease. Fourth, patient-centered humanistic care could increase the compliance of the patients with the HB-GMQ and their satisfaction about the HB-GMQ services. Fifth, patient-centered humanistic care could ensure positive public sentiments toward the outbreak of the pandemic of coronavirus and the HB-GMQ and curb the fearmongering and negative sentiments on social media. Finally, through providing patient-centered humanistic care, the working group had developed growing know-how and insight into addressing key issues related to the management of contagious diseases.

From PKUPH's experience, a sound organizational structure, adequate human and material resources, scientific infection prevention and control, delicate individualized patient care, a complete plan for emergency preparedness, and a multidisciplinary rescue and treatment team constituted the fabric of the HB-GMQ project and determined the final fulfillment of the mission of “no COVID-19 infection and no unanticipated adverse events.”

There is a growing body of evidence showing that most patients undergoing hemodialysis have varying degrees of psychological problems, such as depression and anxiety (Ng et al., 2015). Avdal et al. (2020) also validated that problems of financial losses, social exclusion, stress, fatigue, depression, and anxiety may occur in the patients undergoing hemodialysis and peritoneal dialysis. Besides, the suddenly imposed HB-GMQ might have further aggravated the patients' preexisted psychological problems. Unfamiliar surroundings, misunderstandings toward the HB-GMQ, the fear of the consequences of this infection, and worries over undesirable dietary management in the hospital were all HB-GMQ-induced psychological problems.

Previous studies had revealed that the quarantine did have a psychological impact on patients. During the MERS outbreak, a study conducted by Lee et al. (2018) in the Republic of Korea included 73 hemodialysis patients who were experiencing the hospital-based quarantine. The researchers used the Hospital Anxiety and Depression Scale (HADS) to evaluate the psychological impact of the 2015 MERS on 73 quarantined hemodialysis patients. In the Mini International Neuropsychiatric Interview (MINI), four patients were subjected to major depressive disorder (MDD), and eight patients suffered from generalized anxiety disorder (GAD). In the HADS, eight patients (11%) experienced anxiety, and 11 patients (15.1%) underwent depression (Lee et al., 2018). A recent case of Diamond Princess had also revealed that a 2-week coronavirus quarantine did have a negative psychological impact on the passengers and subjected them to anxiety, depression, and other mental health challenges even after disembarkment (Abrams, 2020). Brooks et al. (2020) illustrated five studies comparing psychological outcomes for people quarantined with those who were not quarantined. The results indicated that a relevant high prevalence of symptoms of psychological distress and disorder was found in those who had been quarantined (Brooks et al., 2020).

In our study, the results showed that no statistically significant differences were found in the anxious or depressive symptomatology of the patients before and during the HB-GMQ. This was probably attributed to the following reasons. First, regular hemodialysis treatment was maintained and provided during the HB-GMQ, which met with some patients' needs for receiving in-patient care and alleviated their worries about the possibility of infection during the commute between their home and the hospital. Second, administrative team, healthcare team, and logistic team were in place to ensure their safety during the HB-GMQ. The administrative team was assigned to do multiple tasks: (1) formulating the generic HB-GMQ plan and emergency plan, ensuring orderly execution of the HB-GMQ plan; (2) coordinating efforts to respond to every emergency; (3) monitoring public sentiments; (4) solving anticipated problems in advance; (5) optimizing management processes and measures; (6) dealing with undesirable emotions and feelings of patients; (7) facilitating personal protective equipment; (8) providing nosocomial infection training; (9) reporting to the authorities; (10) communicating with communities for accessibility, etc. The healthcare team included three subteams, namely, nephrology team, multidisciplinary team and nursing team, who were available 24 h a day to respond to every emergency, and get to know the medical and living needs of the patients and tackle their problems in a quick and appropriate manner. When encountering patients with anxious or depressive symptomatology, the healthcare team would mobilize professionals to provide targeted counseling and therapeutic solutions. In doing so, anxious or depressive symptomatology in the patients could be excluded. The logistic team responded quickly to every arrangement of the generic HB-GMQ plan and formulated the procedures for material acquisition, lift using, infection, cleaning, washing, catering, hospitalization, patient transportation, etc. In order to provide a good environment as much of their home, sanitary, and well-ordered wards were ensured, and high-speed Internet access was guaranteed. After their arrival at the HB-GMQ area, daily necessities, such as toiletries, were purchased and distributed to the patients. To follow a renal diet, the catering staff, under the advice of nephrologists and clinical nutritionists, formulated the dietary plan, and updated the menu every day based on the principles of consuming high-quality protein and limiting fluids, potassium, and calcium. These measures well-exemplified the working staff's efforts in providing patient-centered humanistic care for the quarantined. Third, encouraging and allowing family members to accompany the patients could ameliorate their loneliness during the HB-GMQ. This policy was particularly helpful for older adults who are functionally very dependent on family members. Fourth, expenses of boarding and lodging incurred during the HB-GMQ were completely free of charge for the patients. This practice, to a large extent, had relieved the psychological and economic pressure of the patients and increased that of the level of acceptance of the HB-GMQ.

The main strength of this study is that there is no published research report on the implementation of patient-centered humanistic care for COVID-19 closely contacted hemodialysis patients under the HB-GMQ. As the global COVID-19 pandemic continues, the study could provide a humanistic approach for overcoming a difficult period considering healthcare arrangement and support.

There are also some limitations to this study. First, the intrinsic feature of patient-centered humanistic care lies in its individualized care. The PKUPH's approach to cope with a COVID-19 situation cannot be fully replicated and reproduced by other institutions. Second, the outbreak of the COVID-19 situation in PKUPH was not foreseeable, the knowledge, and perceptions regarding the COVID-19 at that time were very limited, and the suddenly imposed HB-GMQ was not designed—all of these confined the current study to a limited sample size and the specific group of hemodialysis patients. Third, the results may not fully exhibit the whole picture of the psychological impact of COVID-19 on hemodialysis patients and assess the effectiveness of patient-centered humanistic care under the model of patient-centered care of the MMS.

## Conclusion

Throughout the process of the HB-GMQ, the concept and approach of humanistic care under the model of the MMS was highly incorporated into routine management, healthcare, and logistics while adhering to COVID-19 protocols and guidelines of the WHO and Chinese health authorities. PKUPH's experience further validated the hypothesis that patient-centered humanistic care is important in implementing the HB-GMQ and effective in helping the quarantined tide over the HB-GMQ, resulting in a high level of satisfaction about the HB-GMQ, and no symptomatic increase of anxiety and depression in the patients before and during the HB-GMQ. With the concerted efforts from different parties, there have been no unanticipated problems, and no infection cases and no preventable adverse events occurred during the HB-GMQ. According to the patient satisfaction survey, most patients held a positive attitude toward the HB-GMQ with a high level of compliance. At the same time, there was no symptomatic increase of anxiety and depression in the patients by the analyses of the SAS and SDS. It is hoped that PKUPH's experience of implementing patient-centered humanistic care could enlighten other hospitals on how to implement the HB-GMQ and address the HB-GMQ-induced problems.

## Data Availability Statement

The original contributions presented in the study are included in the article/supplementary material, further inquiries can be directed to the corresponding author/s.

## Ethics Statement

The studies involving human participants were reviewed and approved by the Ethical Review Committee of Peking University People's Hospital. The patients/participants provided their written informed consent to participate in this study. Written informed consent was obtained from the individual(s) for the publication of any potentially identifiable images or data included in this article.

## Author Contributions

HC and JW designed the research. HZ, LG, and XL drafted the manuscript. XS, LZ, JG, XH, XJ, JL, ZH, YW, LW, and ZG performed the studies. All authors contributed to writing the article.

## Funding

Peking University Special Fund for COVID-19 Prevention and Control (PKU2020PKYZX001).

## Conflict of Interest

The authors declare that the research was conducted in the absence of any commercial or financial relationships that could be construed as a potential conflict of interest.

## Publisher's Note

All claims expressed in this article are solely those of the authors and do not necessarily represent those of their affiliated organizations, or those of the publisher, the editors and the reviewers. Any product that may be evaluated in this article, or claim that may be made by its manufacturer, is not guaranteed or endorsed by the publisher.
